# Fibroblast and T cells conditioned media induce maturation dendritic cell and promote T helper immune response 

**Published:** 2012

**Authors:** Masoumeh Asadi, Farah Farokhi, Nowruz Delirezh, Meysam Ganji Bakhsh, Vahid Nejati, Keykavos Golami

**Affiliations:** 1 *Department of Biology and Embryology, Faculty of Science, Urmia University, Urmia, Iran; *; 2 *Department of Immunology, Faculty of Veterinary Medicine, Urmia University, Urmia, Iran.*

**Keywords:** Dendritic cell, Fibroblast, T cell

## Abstract

Dendritic cells (DCs) induce pathogen-specific T cell responses. We comprehensively studied the effects of addition of maturation stimulus, fibroblasts (fibroblast conditioned medium), PHA activated T cells (T cell conditioned medium), and mixture of fibroblast & PHA activated T cells (FCM-TCCM) conditioned media on maturation of DCs. Monocytes were cultured with GM-CSF and IL-4 for five days. Maturation factors included MCM and TNF-α as control group. FCM and TCCM, or FCM-TCCM supernatant were considered as the treatment group. Tumor antigens were added at day five. Matured DCs were harvested at day seven. Phenotypic and functional analyses were carried out using anti (CD14, CD80, CD86, CD83 and HLA-DR) monoclonal antibodies. Phagocytic activity, mixed lymphocyte reaction (MLR) and cytokine production were also evaluated. At the end of culturing period, significantly fully matured DCs with large amount cytoplasm and copious dendritic projections were found in the presence of MCM, TNF-α with or without FCM, TCCM, FCM as well as TCCM. Flow cytometric analysis revealed that expression of CD14 decreased in particular in treated DCs, at the 5^th^ day and expression of CD80, CD86 and HLA-DR was higher when FCM, TCCM, FCM plus TCCM were added to maturation factor. This study demonstrated that DCs matured with these methods had optimum function in comparison with either factor alone.

## Introduction

So far much attention has been paid to immuno-therapeutic strategies that are used to induce antitumor immunity in cancer patients as a novel tumor cell-specific therapeutic approach in the last decade.^1^ Among these therapeutic methods, DC-based vaccines are currently regarded as powerful vaccination protocols. DCs are the most potent leukocyte populations which control the primary immune response. Antigen-presenting competent cells recognize and process antigens in the peripheral blood and tissues migrate to draining lymph nodes. These cells finally present antigens to the target resting lymphocytes. Antigens are very efficiently internalized and processed by immature dendritic cells and presented when they acquire matured phenotype through migration from tissue to sentinel lymph node.^2^ Maturated Dendritic cells reduce antigen uptake capacity and increase co-stimulatory molecule expression and secretion of various cytokines.^3^^,^^4^ Monocyte conditioned medium (MCM) is generated in vitro by culturing monocytes and then using the culture supernatant fluid as a source of maturation factors. Other maturation protocols are reported in other studies. Like TNF-α without any supplement,^5^ or in combination with other factors like prostaglandin E2 (PGE2)^6^ vasoactive intestinal peptide,^7^ poly-I-C^8^ bacterial lipopolysaccharide (LPS),^9^ as well as mycobacterium or components of mycobacterium.^10^^-^^12^

During migration from peripheral, DCs are impressed with stromal microenvironment which is composed of the extracellular matrix, soluble mediators and variety of different cell types. Stromal cells such as fibroblasts and endothelial cells display different functional properties and exert numerous modulatory effects on DC maturation during its maturation.^13^^-^^14^

Fibroblasts are extremely heterogeneous multi-functional cells that their role in wound healing, developmental processes and tumor development is well established.^15^ However, fibroblasts are capable of producing various paracrine immune modulators such as peptide growth factors, cytokines, chemokines and inflammatory mediators.^16^ They express cell surface and co stimulatory molecules such as CD40. It’s believed that fibroblasts could actively participate in the regulation of inflammation and immune responses.

Soluble proteins released from activated T cells may contribute to immune activation.^17^^-^^18^ For example, TNF-α is a protein that can exist as a soluble molecule or as a membrane-associated glycoprotein in activated T cells. Similar to TNF-α, it is recently found that activated CD4^+^ T cells not only express the membrane form of CD40L but also produce a soluble form, sCD40L, *in vitro* and *in vivo*. Importantly, cell-free supernatants from activated T cells contain sCD40L. This supernatant can induce CD40-bearing cells to express enhanced levels of the important costimulatory molecules, CD80, CD86. A study indicates that sCD40L has a biological activity.^19^ In the present study, effects of FCM or TCCM or a mixture of them was examined on the maturation of DCs. 

## Materials and Methods

**Media and reagents. **Complete medium (CM) including RPMI-1640 (Gibco, Eggenstein, Germany) supplemented with 10% human AB serum (Blood Transfusion Organization, Tehran, Iran), 2.5 × 10^-5^ M 2ME, 2 mM L-glutamine (Sigma Chemical Co., Munich, Germany), 100 U mL^-1^ penicillin and 100 µg mL^-1^ streptomycin (Sigma Chemical Co., Munich, Germany) was used. Recombinant human GM-CSF (Novartis AG, Basel, Switzerland), IL-4 (Sigma Chemical Co., Munich, Germany), tumor necrosis factor alpha (TNF-α) (Peprotech, London, UK). MCM were used to derive DC from peripheral blood monocytes. Phytohemagglutinin (PHA) (Sigma Chemical Co., Munich, Germany) and IL-2 (Sigma Chemical Co., Munich, Germany) Allogenic for activate T cells. Mixed leukocyte reaction (MLR) was performed by [H^3^] thymidine (Amersham Pharma, London, UK) uptake test. Finally, interferon gamma (IFN-γ) and IL-4 cytokines release assay were performed using commercially available ELISA kits (R & D Co., Stockholm, Sweden). Phagocytic activity was measured using fluorescent isothyocyanate (FITC) labeled latex beads (Sigma Chemical Co., Munich, Germany) and cell surface activity was quenched by quenching buffer (NaCl 0.9%, citrate buffer 13µM, and trypan blue 0.25 mg mL^-1^ (all from sigma Chemical Co., Munich, Germany).

**Cell Lines and conditioned media. **Human skin fibroblast (HSFPI3) and K562 erythroleukemia cell lines were purchased from National Cell Bank of Iran (NCBI, Tehran, Iran). HSFPI3 was cultured in CM supplemented with 10% FCS to reach 80% confluency. The supernatants then were replaced with fresh medium without serum and incubated for 48 hours. The conditioned media were ﬁltered through 0.22 µm ﬁlter, and stored at -80 ˚C.T cells were cultured in the presence of IL-2 (20 U mL^-1^) and PHA (20 µg mL^-1^). Monocyte and PHA activated T cells conditioned medium (MCM and TCCM), the supernatant of overnight culture of autologous adherent and non-adherent PBMCs were also ﬁltered and stored as mentioned above.

**Preparation of tumor cell lysate. **2 × 10^7^ K562 cells were washed in CM and subjected to four freeze (liquid nitrogen) and thaw (37 ˚C water bath) cycles to obtain a crude lysate. Then removal of large particles was used by centrifuge (2000 *g*, for 10 min followed by 13000 *g*, 60min, at 4 ˚C). After filtering through a 0.22 µm mesh, the protein content was determined by the Coomassie blue protein assay (Biorad, London, UK) and aliquots were stored at – 80 ˚C.

**Generation of tumor cell lysate pulsed DCs. **Monocyte derived DCs were generated as described by others.^20^ Peripheral blood mononuclear cells (PBMCs) from blood donors was isolated on lymphoprep (1.077 g mL^-1^ Nycomed Pharma, Oslo, Norway). Blood was drawn from donor into a heparinized blood tube. Blood was diluted with RPMI at room temperature in a conical tube. Diluted blood was with Ficoll Hypaque lymphocyte separation medium and centrifuged at 2500 rpm without brake at room temperature for 20 minutes. Buffy coat was removed and transferred into a new conical tube. RPMI medium was raised and spun at 2000 rpm at room temperature for 10 minutes. Cells were washed by re-suspending pellet in RPMI and spinning at 1200 rpm at room temperature for 10 minutes. PBMC was re-suspended in CM supplemented with 10% AB serum and cultured in T25 flask for 2 hours at 37 ˚C. The non-adherent cells were removed and adherent cells were cultured in CM containing GM-CSF (1000 U mL^-1^) and IL-4 (800 U mL^-1^). Cultures were fed removing 2 mL of medium and adding 3 mL fresh medium with cytokines every other day. At day 4 of experiment tumor cell lysate was added to DC to final protein concentration of 50 µg mL^-1^ and incubated overnight. Maturation factors, including MCM (25% v/v), TNF-α (10 ng mL^-1^) were considered as control group. Skin fibroblast supernatant (25% v/v), PHA activated T cells supernatant (25% v/v), or mixture of skin fibroblast and PHA activated T cells supernatant (50% v/v) were added to the groups 1 to 3, respectively, at day five. Tumor antigen pulsed matured DCs were then harvested at day seven.^21^

**Antibodies and flow cytometry. **Immunophenotyping of monocyte derived DCs was performed by direct immunofluorescence staining of cell surface antigens using FITC or RPE conjugated mouse antibodies against, CD14, CD80, CD83, CD86, HLA-DR, and appropriate isotype matched controls (Serotec, London, UK). Samples were analyzed on Dako flow cytometry system (Partec, Münster, Germany). The data from recent experiment were analyzed by Flow Max software.

**Allogenic mixed leukocyte reaction (MLR). **MLR assay was performed using tumor lysate pulsed autologous DCs which were irradiated to 3000 rad as stimulator and allogenic peripheral blood mononuclear cells (PBMCs) as responder cells in the ratios of 1:5, 1:10 and 1:20. A phytohemagglutinin stimulated T cells (2.5%) (Sigma Chemical Co., Munich, Germany), and DCs and PBMCs alone were served as positive and negative controls, respectively. Cultures were made in V bottom 96 well plates at final volume of 200 µL of CM supplemented with 10% AB serum for 5 days. In this experiment the [^3^H] thymidine was added at concentration of 1µCi/well at least 18 hours before beginning of rearing. Proliferative responses were measured by a liquid scintillation counter (Wallac Inc., Turku, Finland). The data were expressed as mean count per minute and stimulation index (SI) obtained for triplicate wells.^22^

**Phagocytosis assay. **Mature DCs were subjected to phagocytosis assay at day seven. FITC conjugated latex beads were opsonized by 10% human AB serum at concentration of 2.5 × 10^8^ beads mL^-1^ for 30 minutes at room temperature. Twenty micro liters of opsonized beads were added to 250 ×10^5^ DCs and incubated for 48 hours at 37 ^o^C and 5% CO_2_. The cells were then harvested and washed with quenching buffer three times (300 *g* for 10 min). Phagocytic activity was analyzed in terms of percentage and mean fluorescence intensity (MFI) of positive cell on Dako flow cytometry system (Partec, Germany) and FlowMax software.

**Cytokine assay. **Supernatants of mature DCs at day seven were subjected to measure IL-10 and IL-12. Supernatant of MLR cultures at day five (before addition of [^3^H] thymidine), were collected to measure IFN- γ and IL-4 release. Cytokine was assayed commercially using available sandwich ELISA kits as instructed in catalogue (R & D Co Stockholm Sweden). Cytokine release was reported as mean ± SEM for duplicate wells.^21^

**Statistical analysis. **Data were analyzed using Student *t-*test. Values of *P *< 0.05 were considered significant. 

## Results

**Fig. 1:**
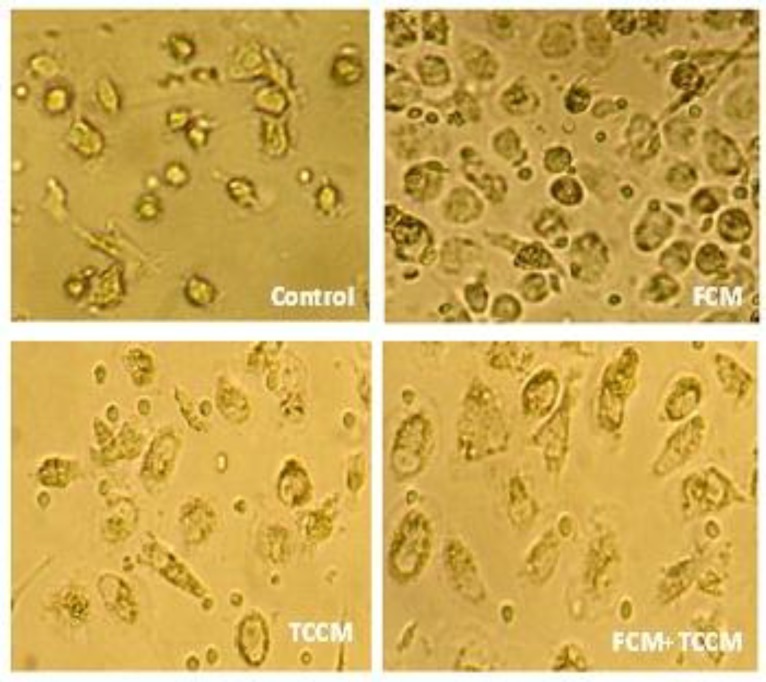
Monocyte-derived DCs. Control: DCs differentiated in the presence of MCM, TNF-α. FCM: DCs differentiated in the presence of MCM, TNF-α, FCM. TCCM: DCs differentiated in the presence of MCM, TNF-α, TCCM. FCM-TCCM: DCs differentiated in the presence of MCM, TNF-α, FCM-TCCM. The most of mature DCs were appeared as a single cells or loosely adherent aggregates reviewing by light microscopy. (400×).

**Morphology. **Dendritic cells (DCs) were generated from peripheral blood monocytes of volunteers in four setup groups. In control group MCM and TNF-α was added as conventional maturation factor and in three test groups FCM, TCCM and FCM plus TCCM were added to MCM and TNF-α to induce extra maturation. Three days after culturing plastic adherent monocytes in the presence of GM-CSF and IL-4, clusters of non-adherent cells were appeared and increased in size and number thereafter. Maturation factors were added to immature DCs pulsed with tumor cell lysate at day 5 and 60–70% of cells was appeared to loosely adhere to each other in clumps or was isolated floating cells with typical dendritic morphology by day seven. At this stage we found no differences (*P*>0.05) between FCM, TCCM or both treated and untreated DCs of all three groups exhibited same typical cytological features, i.e., large irregular cells with numerous cell membrane processed under light microscopy (Fig. 1).

**Fig. 2:**
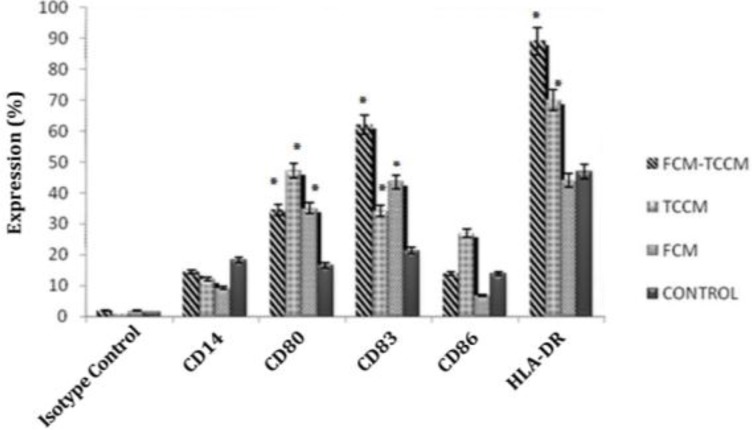
Flow cytometreic analysis of expression of CD14, CD80, CD86, HLA-DR and CD83. Monocyte-derived DCs differentiated in the presence of MCM, TNF-α, with or without FCM, TCM, FCM plus TCCM were harvested on day 7 and analyzed by using respective monoclonal antibodies and isotype controls. Data represent the mean ± SD of three independent experiments

**Fig. 3:**
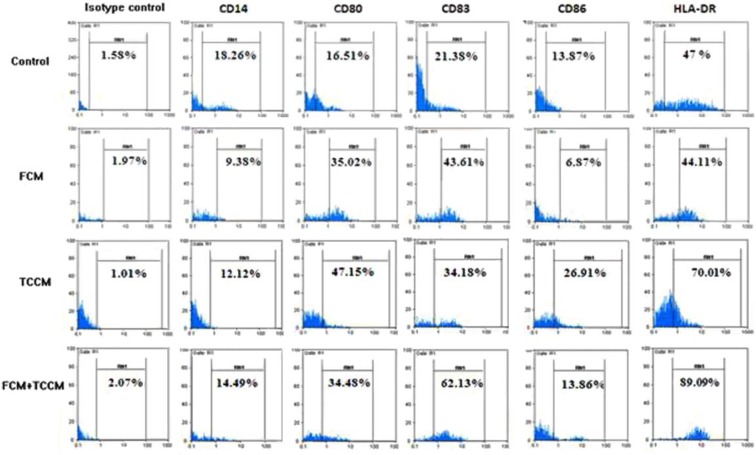
Changes in surface-antigen phenotype of DC treated with FCM, TCCM and FCM+TCCM. Human peripheral blood monocytes were cultured with GM-CSF and IL-4 for 7 d and further with medium alone (first), FCM (second), the TCCM of PHA- activated T cells (third), FCM+TCCM (forth). DC were examined for surface expression of HLA-DR, CD86, CD83, CD80 and CD14 by flow cytometry. Incorporation of FITC-conjugated dextran (F-DEX) was also assessed by flow cytometry.

**Phenotype. **Flow cytometric analysis of DCs revealed significant differences (*P*<0.05) in the expression of surface molecules crucially involved in DC functions. Compared to control group, FCM, TCCM, FCM plus TCCM treated DCs consistently showed a substantially enhanced expression of HLA-DR, CD80, CD86 and CD83 and, decreased expression of CD14 (Fig. 2). The results showed that addition of FCM and TCCM to MCM plus TNF-α were more potent than either factor alone to induce expression of maturation and co-stimulatory markers (CD83, HLA-DR, CD80 and CD86) (Fig. 3). Altogether our results showed that treatment of monocytes with FCM, TCCM, especially FCM plus TCCM at day 5 and along with other maturation factors will lead to generation of more potent mature DCs.

**Fig. 4:**
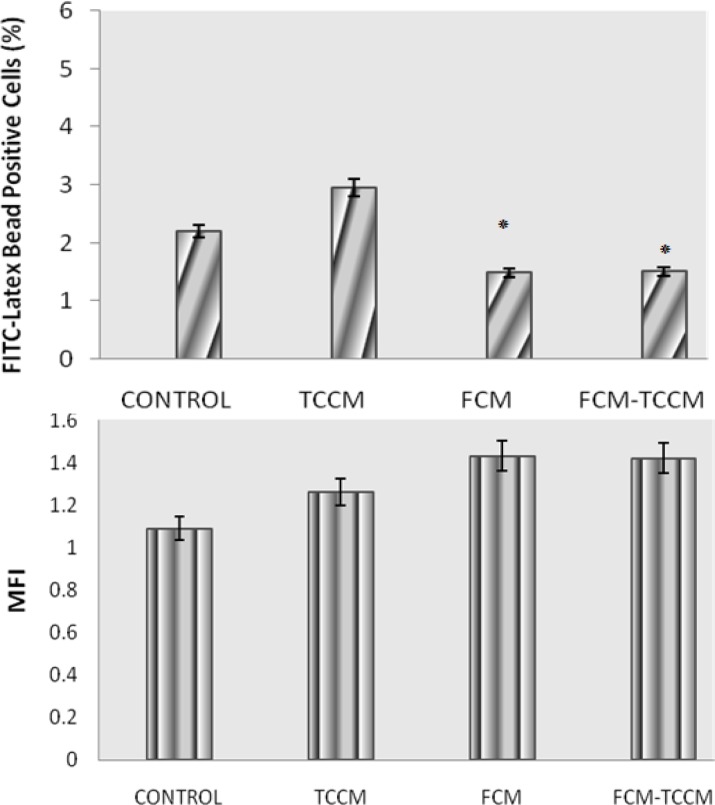
DC phagocytic activity. Phagocytic activity of MCM, TNF-α, with or without FCM, TCM, FCM plus TCCM treated immature and mature DCs was measured using FITC-conjugated bead uptake and results of percent and mean fluorescent intensity (MFI) of phagocytic cells. Data represent mean ± SD of three independent experiments. * indicates significant differences at *P *< 0.05.

**Phagocytic activity. **Dendritic cells maturation process results in substantially decrease in phagocytic activity. Comparison of phagocytic activity between FCM, TCCM FCM plus TCCM treated DCs and controls at day 7 (mature DCs) revealed that FITC-conjugated latex bead engulfment was significantly decreased during maturation process (*P* ≤ 0.05). Intergroup comparison showed that in spite of decrease in latex bead positive cells in FCM, TCCM FCM plus TCCM treated DCs, mean fluorescent intensity of these cells has been increased (Fig. 4).

**Fig. 5:**
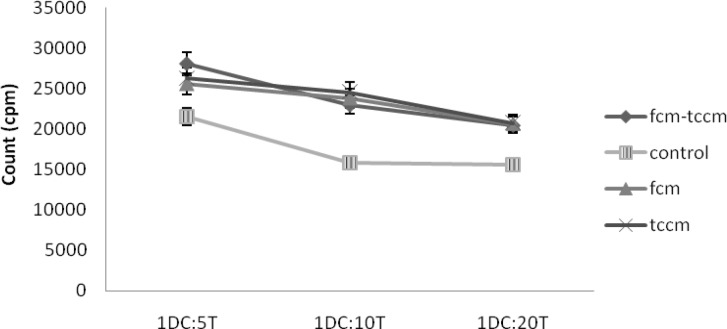
Cytokine release from allogenic MLR. Supernatants of MCM, TNF-α, with or without FCM, TCM, FCM plus TCCM matured DCs as well as of allogenic MLR, IFN-γ and IL-4 cytokines assay, respectively. The results are expressed as a mean of triplicates

**Mixed leukocyte reaction (MLR). **Tumor antigen pulsed matured DCs would be able to induce a proliferative response in allogenic MLR. No adherent PBMCs were stimulated with DCs pulsed with tumor lysate at a ratio of 1:5, 1:10, and 1:20. Our results showed that T cell proliferative responses were elicited in three respective ratios for all four examined groups. However, proliferation rate was higher in FCM, TCCM, FCM plus TCCM \treated groups control group but it was non-significantly (*P* ≤ 0.05) (Fig. 5).

**Fig. 6:**
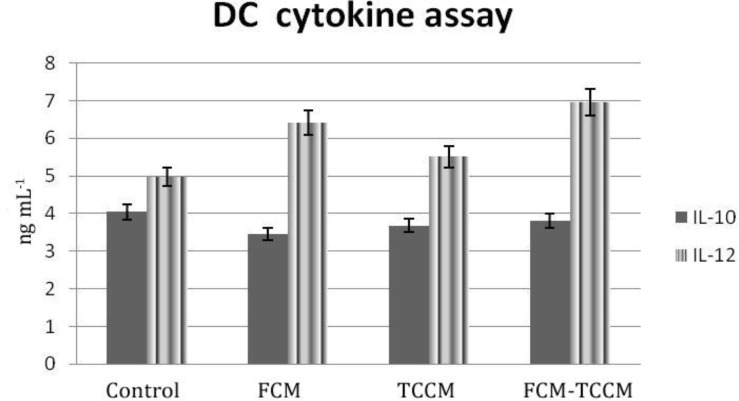
Cytokine release from DCs. Supernatants of MCM, TNF-α, with or without FCM, TCM, FCM plus TCCM matured DCs as well as supernatants were subjected to IL-12, IL-10cytokines assay respectively. The results are expressed as the mean of triplicates.

**Fig. 7:**
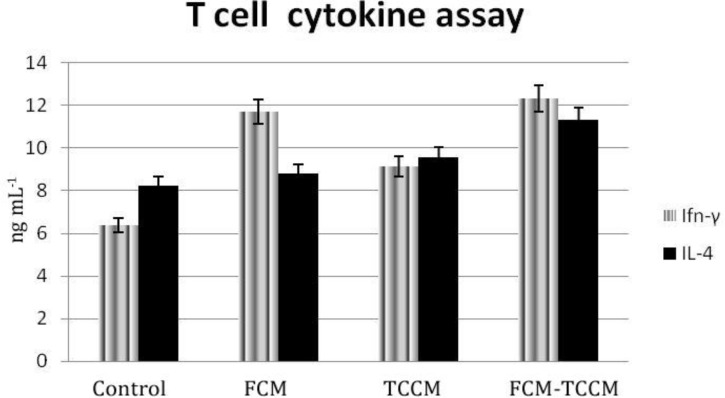
Cytokine release from Allogenic MLR. Supernatants of MCM, TNF-α, with or without FCM, TCM, FCM plus TCCM matured DCs as well as of allogenic MLR IFN-γ and IL-4 cytokines assay respectively. The results are expressed as the mean of triplicates.

**Cytokine release**. It has been reported that antigen pulsed DCs could secrete cytokines upon maturation in the presence of various maturation factors. Polarization of DCs to either DC1 or DC2 would result in antigen specific TH1 or TH2 responses, respectively. To address this issue, we examined cytokine profiles produced by antigen pulsed DC and respective primed T cells in the supernatant of mature DCs and MLR reaction DCs in all examined groups released variable amounts of either IL-12 or IL-10 (Fig. 6). IL-12 was released in higher amount than IL-10, so the ratio of IL-12: IL-10 was increased in test groups particularly in FCM, FCM plus TCCM treated ones (Fig. 6). Primed T cells produced either IFN–γ and IL– 4 in response to stimulation by DCs. IFN–γ was released in higher amount in test groups compared to the control. Although both cytokines were produced in higher level by FCM plus TCCM treated group, IFN–γ: IL– 4 ratio was highest in FCM treated group. Interestingly, IFN–γ and IL-4 production by primed T cells were well correlated to the levels of IL-12 and IL-10 produced by DCs (Fig. 7).

## Discussion

Fibroblasts and T cells should be considered as active contributors to the regulation of the inflammatory response. Meanwhile, it is generally believed that dendritic cells are potent inducers of CD4^+^ and CD8^+^ T cell mediated responses. Moreover, evidences suggest that tumor antigen loaded DCs may yield enhanced antitumor immunity *in vitro* as well as *in vivo*.^23^

DCs in peripheral blood is not high enough to be used in experimental or clinical settings, instead they generate large numbers of DCs from either bone marrow derived CD34^+^ precursors or peripheral blood monocytes.^24^^,^^25^ Given unique properties and potent ability of *in vitro* generated matured DCs to stimulate naive T cells, it is not surprising that different DC maturation protocols have already been applied to *in vitro* DC generation.^24^^,^^26^ In the present study peripheral blood monocyte derived dendritic cells were loaded with tumor antigens and matured in the presence of FCM, TCCM, FCM plus TCCM in addition to MCM and TNF-α was used to analyze their phenotypic and functional properties. 

Previous studies^27^ as well as our experience^28^ have described the matured DCs as non-adherent, large irregular veiled cells with numerous cell membrane processes. Morphological examination of floated cells using inverted or phase-contrast light microscopy after treatment with FCM, TCCM, FCM plus TCCM at day 7 of culture revealed no differences among these four kinds of maturation protocols (Fig. 1). So we precede the work with comparison of phenotypic and functional properties. CD14 and CD83 are the main cell surface markers that characterize *in vitro* generation of immature and matured DCs from peripheral blood monocytes, respectively. In addition, CD80 and CD86 are known as co-stimulatory molecules which participate in activation of T lymphocyte responses.^29^ In the present study we used these markers to compare the phenotype of DCs generated in the presence of FCM, TCCM, FCM plus TCCM and/or MCM plus TNF-α. It is well known that *in vitro* conversion of peripheral blood monocytes to immature DCs was accompanied by down regulation of CD14 expression,^5^ and the reduction in expression of CD14 was appeared when FCM, TCCM, FCM plus TCCM were added to DCs at day 5 of the culture, respectively. TNF-α and MCM have the least effect on elimination of CD14 expression. However, MCM plus TNF-α and poly (I-C) had the least effect on elimination of CD14 expression.

It is noteworthy, treatment of DCs with FCM-TCCM, FCM and TCCM resulted in higher expression of CD80, CD86, CD83 and HLA-DR in comparison to control group (Fig. 2). Altogether our results showed that treatment of monocytes with FCM-TCCM at day 5 and along with other maturation factors led to generation of more potent mature DCs. The choice of a maturating agent for DC should not only be based on its impact on cell-surface phenotype but should also take into account the nature of the functional state of DC, which have an instructive role for the immune response including auto or allo mixed lymphocyte reaction, phagocytic activity and cytokines released from DC stimulated T lymphocytes. It is well known that phagocytic activity of DCs is decreased during maturation process,^27^ so in the present study we examined phagocytic activity of FCM, TCCM, FCM plus TCCM treated and untreated matured DCs. Based on previous reports, we found that FITC-conjugated bead uptake by DCs treated as well as control DCs was decreased during maturation period. In spite of significant decrease in the percent of FITC-conjugated bead positive cells, mean fluorescent intensity (MFI) of these cells was increased in matured DCs. Furthermore, treatment of the monocytes with fibroblast and T cell conditioned medium also increased MFI (Fig. 4). Altogether, our repeated experiences with this and other maturation factors showed that maturation process decreased the number of phagocytic cells, but few cells which have still their own phagocytic capacity, uptake much more beads and express higher MFI values. It is reported that DCs matured with various maturation factors such as MCM, poly I-C, cytokines cocktail as well as heparin are powerful stimulator of MLR,^29^^,^^30^ so, in this study we compared the potential of FCM, TCCM, FCM plus TCCM and/or MCM plus TNF-α treated DCs in stimulation of allogenic lymphocytes in the ratios of 1:5, 1:10 and 1:20. We found that it was not significantly different if fibroblast and T cell conditioned medium or their mixture to DC culture are added. All treatment groups were capable to induce allogenic MLR in all three examined ratios, however, addition of FCM, FCM-TCCM and TCCM to MCM plus TNF-α and poly (I-C) augmented their stimulatory capacity insignificantly (Fig. 5).

The strategy of DC maturation and antigen loading clearly influences the ability of DC to polarize T cell for TH1/TH2 response and determines the outcome of elicited immune response.^31^ In this study, we evaluated IL-12 and IL-10 produced by matured DCs, IFN-γ, and IL-4 cytokine were produced by primed T cells as representatives of type 1 and type 2 cytokine patterns, respectively. 

We examined cytokine profiles produced by monocyte derived DCs and respective primed T cells in the supernatant of mature DCs and MLR reaction. DCs in all examined groups released variable amounts of either IL-12 or IL-10. IL-12 in higher mounts in three treated groups than control, but IL-10 was released in fewer amounts in three treated groups than control (Fig. 6). The ratio of IL-12: IL-10 was higher in group FCM-TCCM. Primed T cells produced either IFN–γ or IL–4 in response to stimulation by DCs. Both cytokines were released and IFN–γ: IL-4 ratio was higher in the DCs treated group rather than control group. Interestingly, IFN–γ and IL-4 production by primed T cells was well correlated to the levels of IL-12 and IL-10 produced by DCs. According to this study Mixture of FCM and TCCM may help polarization of TH1 (Figs. 6 and 7).

A review of literature showed that an *in vitro* system of co-culture of CD34+ cord blood cells with cutaneous fibroblast cell lines supported the development of dermal DC,^22^ indicating that specific stromal elements within dermis could regulate DC development. Reportedly, human DC generated from peripheral blood monocytes specifically interact with human dermal fibroblasts via the interaction of 2 integrins on DC with Thy-1 (CD90) and ICAM-1 on fibroblasts.^22^ This induced the phenotypic maturation of DC reflected by expression of CD83, CD86, CD80, and HLA-DR in a TNF-α and ICAM-1-dependent manner. Moreover, fibroblast-matured DC potently induced T cell activation reflected by CD25 expression and enhanced T cell proliferation.^32^ It is reported that fibroblast conditioned medium (FCM) potently inhibits the maturation and expression of major histocompatibility complex class II and co-stimulatory molecules induced by stimulation of spleen-derived DC As fibroblasts are present in the tissue microenvironment. They are active players in the establishment of an immune response and the nature and role of the fibroblastic inhibitory activity remain to be established.^33^

Reddy *et. al* reported MCM importance for immature DC to become active because it contains several cytokines including TNF-a, IL-1b, IL-6, and IFN-gamma.^26^ Active DCs are effective at stimulating the proliferation of naive CD4 T cells but seldom secrete IL-12 as a key cytokine for the differentiation of Th1 cells.^26^ Regarding MCM result we were struck with the idea that supernatant from activated T cells might be a strong candidate for inducing terminal maturation of DC, although it has been previously reported that culture supernatants of some tumor cells or regulatory T cells contain IL-10 that are capable to decrease expression of co stimulatory molecules, secretion of IL-12 and inhibition of DC maturation and function.^30^^,^^31^ Observation of Kazunori Kato demonstrated T cells super-natant contains high levels of biological active forms of CD40L, TNF-α, and IFN-gamma. Activated T cells supernatant (TCCM), because of containing TNF-α and IFN-γ, is able to involve in the up-regulation of adhesion molecules and co stimulatory molecules on a variety of tumor cells.^34^ sCD40L or an sCD40L chimeric protein can induce cytokines (i.e., IL-12, IFN-α, and TNF-α) and APC to express immune accessory molecules (i.e., CD80, CD83, and CD86) in a manner similar to that of the membrane-bound form of CD40L on activated T.^35^^-^^37^ For enhancing the maturation of CD11c1 myeloid DC, -g was the most potent cytokine. Finally recent studies demonstrated that T cell–conditioned media (TCCM) have the capacity to differentiate monocytes into functional DCs, a process substantially mediated by T cell-derived CD40L, TNF-α, and IFN-gamma.^34^ As mentioned above it is obvious that cytokines released from Fibroblast and T cells induced the phenotypic and functional maturation of DCs which not only was reflected by expression of CD83, CD86, CD80, and HLA-DR but by decreased Ag endocytosis and facilitated the differentiation of T cells and polarization of immune response. In consistent with these results, we found that generation of monocyte–derived DCs in the presence of FCM,TCCM, either FCM plus TCCM at day 5 induced enhanced expression of co-stimulatory molecules

(CD80 and CD86 and low CD83) as well as HLA-DR and decreased phagocytic activity. In this setting DCs developed toward DC1 and induced T cells to TH1 type cytokine release (Enhanced levels of IL-12: IL-10 and IFN-γ: IL-4 ratios, respectively).

In conclusion, since DCs play a pivotal role in immune responses, a great deal of research effort has been focused on the potential clinical application of these cells in immune therapeutic settings. Our findings indicated that the yields, viability, and morphology of MCM, TNF-α and added conditioned medium. Dendritic cells in 3 treatments especially mixture FCM with TCCM may be more efficacious than DCs treated with MCM, TNF-α for the induction and polarization of immune responses. Because more maturation agents considered for clinical DC-based vaccination protocols are monocyte conditioned medium (MCM) and TNF-α^21^ addition of FCM and TCCM especially a mixture of FCM and TCCM can easily be adapted for the generation of large amounts of mature DCs from monocytes and considered as reliable approach for immunotherapy.
